# How Can Selection of Biologically Inspired Features Improve the Performance of a Robust Object Recognition Model?

**DOI:** 10.1371/journal.pone.0032357

**Published:** 2012-02-27

**Authors:** Masoud Ghodrati, Seyed-Mahdi Khaligh-Razavi, Reza Ebrahimpour, Karim Rajaei, Mohammad Pooyan

**Affiliations:** 1 Department of Biomedical Engineering, Faculty of Engineering, Shahed University, Tehran, Iran; 2 School of Cognitive Sciences (SCS), Institute for Research in Fundamental Sciences (IPM), Niavaran, Tehran, Iran; 3 Brain and Intelligent Systems Research Laboratory (BISLab), Department of Electrical and Computer Engineering, Shahid Rajaee Teacher Training University, Tehran, Iran; 4 Department of Mathematics, Statistics and Computer Science, University of Tehran, Tehran, Iran; 5 Department of Mathematics and Computer Science, Amirkabir University of Technology, Tehran, Iran; Heidelberg University, Germany

## Abstract

Humans can effectively and swiftly recognize objects in complex natural scenes. This outstanding ability has motivated many computational object recognition models. Most of these models try to emulate the behavior of this remarkable system. The human visual system hierarchically recognizes objects in several processing stages. Along these stages a set of features with increasing complexity is extracted by different parts of visual system. Elementary features like bars and edges are processed in earlier levels of visual pathway and as far as one goes upper in this pathway more complex features will be spotted. It is an important interrogation in the field of visual processing to see which features of an object are selected and represented by the visual cortex. To address this issue, we extended a hierarchical model, which is motivated by biology, for different object recognition tasks. In this model, a set of object parts, named patches, extracted in the intermediate stages. These object parts are used for training procedure in the model and have an important role in object recognition. These patches are selected indiscriminately from different positions of an image and this can lead to the extraction of non-discriminating patches which eventually may reduce the performance. In the proposed model we used an evolutionary algorithm approach to select a set of informative patches. Our reported results indicate that these patches are more informative than usual random patches. We demonstrate the strength of the proposed model on a range of object recognition tasks. The proposed model outperforms the original model in diverse object recognition tasks. It can be seen from the experiments that selected features are generally particular parts of target images. Our results suggest that selected features which are parts of target objects provide an efficient set for robust object recognition.

## Introduction

How different objects are recognized in the visual cortex has been a challenging and major question in the field of vision neuroscience and machine vision. The visual system of humans and other mammals can simply and rapidly recognize a wide variety of objects in various conditions such as changes in size, position, illumination, viewpoint, etc. in a natural scene. They can even detect and recognize a specific object in a cluttered scene without consuming noteworthy amount of time and effort unlike the best machine vision systems. Achieving a model which can emulate this remarkable system with such a high performance is a long-time goal in computational neuroscience. Although presenting a model with a high performance in object recognition tasks is a goal of interest, plausibility with the primate visual system has much more significance, particularly in the recent decades. A large number of object recognition models have been introduced up to now and ,interestingly, a vast majority of them have shown to perform successfully in different object recognition tasks [1]–[5]. Nonetheless, a very few models are consistent with psychophysical and physiological data throughout the different areas of the visual system [6]–[10]. Furthermore due to the complexity of the human visual system, constructing biologically plausible object recognition models is so difficult.

The first model that qualitatively described simple and complex cells in the primary visual cortex in non-human primates was introduced by Hubel & Wiese [11], [12]. They described a hierarchy of cells in the primary visual cortex. Briefly, their model starts by radially symmetric cells which respond to a spot light (like Lateral Geniculate Nucleus cells, LGN) and alternates by simple cells that respond to bars or edges stimuli at a particular orientation, position and phase within their receptive fields. The next stage of hierarchy is complex cells, they respond well to oriented bars or edges anywhere within their receptive fields and are not sensitive to both location and the phase of the bar. The final stage of the hierarchy is hypercomplex cells which are not only invariant to the position and phase of the bar; they are also selective for the length of the bar. Since then, by their pioneering work, a large number of hierarchical models of the visual cortex have been developed. One successful model which mimic the hierarchical structure of the visual cortex is Neocognitron. It was shown to perform extremely well in the field of digit recognition [6], [13]. Another model of the visual system that is constructed of several layers of Self-Organizing Map (SOM) networks is VisNet [9]. The VisNet has been shown that is able to develop view-invariant representations of the individual synthesic objects [14]. A more complex model of the visual cortical circuits is LAMINART that models the neural circuits in the visual system at an inimitable level of details [7], [15].

By now, very few numbers of models of the visual system have been extended to deal with a variety of real-world image databases [10], [16], [17]. One biologically motivated model in this among is the HMAX model which firstly proposed by [10] and then extended by [17] (http://cbcl.mit.edu/software-datasets/index.html). This hierarchical model of the visual system is based on Hubel & Wiesel model and has tried to quantitatively model the visual ventral pathway during visual processing and object recognition tasks based on recent neurophysiological and psychophysical evidence (Visual information in the ventral pathway is conducted from the retina to the LGN, then to primary visual cortex, V1, V1 sends projections to higher visual areas V2 and V4, the projections from V4 are sent to the last visual area along the ventral stream, inferior temporal cortex, IT. There are projections from IT to prefrontal cortex, PFC; this area is associated with perception, memory and action) [18], [19]. The HMAX model has exhibited outstanding performance on a variety of different object categories. It has also shown to be able to learn features from very few training examples with no prior knowledge [17]. In its simplest architecture, the HMAX model consists of a hierarchy of four layers of computational units (*S_1_*, *C_1_*, *S_2_* and *C_2_*) in order to, firstly, increase specificity and ,secondly, invariance along the hierarchy. The simple *S* units alternate with complex *C* units. The *S* units combine their inputs with Gaussian-like tuning to increase object selectivity and build more complex features from simple ones, while *C* units perform a nonlinear MAX pooling operation over units tuned to the same feature but at different positions and scales to make the response more invariant to translation and scale (more explanation of the HMAX model is described in Materials and Methods section). During training stage in the HMAX model, a large number of image parts, named patches, of various sizes and at random positions are extracted from a training set of images at the level of the *C_1_* layer for all orientations (0°, 45°, 90°, 135°),(i.e., a patch *P* of size n*n contains n*n*4 elements –n varies from 4 pixel to 16 pixel by step 4). This pool of patches has an important role in training process and finally in the task of recognizing different objects.

Several studies have been done to select patches which are more informative than randomly extracted patches by the HAMX model. T. Serre et al. [20], found that the original HMAX model [10] failed to recognize faces in cluttered background and this led to a poor recognition performance. They used a clustering algorithm such as K-means to learn object class-specific visual features of intermediate complexity in order to improve the performance of the model in the task of face detection. E. Meyers et al. [21], used the HMAX model for face processing and modified the standard model in order to create a new set of features which is useful for face identification and finally achieved a higher performance in this specific task. Their major modification to the HMAX model was a linear combination of the *C_1_* outputs to build face identification specific features. They used kernelized and regularized version of the relevant component analysis algorithm (KR-RCA), to obtain the linear combination weights from a training set of images. Although using the KR-RCA algorithm to find the linear combination weights may not be biologically plausible, the performance level was significantly improved. E. Krupka et al. [22] proposed a method which tried to learn to select high-quality features from its properties. They tested their algorithm on the standard model of HMAX. For this purpose, they assumed that each feature is described by a set of properties and they suggested a new algorithm called Meta-Feature based Predictive Feature Selection (MF-PFS) which uses predicted quality to select new good features, while omitting many low-quality features. They eventually compared their results with another selection method, Recursive Feature Elimination (RFE) and reported improvement in the performance.

Evolutionary algorithms such as genetic algorithms play a considerable role in feature selection [23]–[26] and system optimization [27]–[31]. They are widely employed to select a subset of informative features for the purpose of attaining to a higher classification rate. In our study we incorporate Genetic Algorithm (GA) with the biologically motivated hierarchical model, HMAX, in order to select optimized features in the learning stage. Biologically evidence suggests that both genetic factors and visual experience at the time of developing and after that can determine the connectivity and functional properties of units [32]–[34]. It is assumed that the learning plays a key role in determining the wiring and the synaptic weights for the S and the C layers. The proposed model uses these optimized features in different object recognition tasks and successfully achieves a high recognition performance. To test the proposed model, we use various image data sets from the well-known CalTech101 (http://www.vision.caltech.edu/Image_Datasets/Caltech101) [4], CalTech5 [1] from CalTech image data set (http://www.robots.ox.ac.uk/~vgg/data/data-cats.html) and GRAZ-01, GRAZ-02 [35] from the PASCAL Object Recognition Database Collection (http://pascallin.ecs.soton.ac.uk/challenges/VOC/databases.html) and compare our results with [17], [35], [36]. As the results represent, the proposed model is completely task independent unlike [20], [21] and outperforms the HMAX model in different object recognition tasks as well as Moment Invariants, SIFT, SM, and Basic moment in [35]. It also outperforms the EBIM in some cases [36].

## Materials and Methods

### The biologically motivated object recognition model

The standard HMAX model is based on the hierarchical theory of visual processing and its architecture is derived from the well-known model of Hubel & Wiesel [11], [12]. It models the ventral visual pathway from V1 (the very first processing part in the visual cortex) to higher levels of visual cortex such as IT cortex and PFC. The first processing units in the HMAX model (*S_1_*, C1) play the role of simple and complex cells in the Hubel & Wiesel model which are consistent with the cells in V1. The simple cells are selective to a bar (edge) with a specific position and orientation in their receptive fields. The complex cells receive their inputs from several simple cells so they can easily respond to bars in different positions and orientations within their receptive fields. The combination of these two types of cells builds up the position and size invariance properties. The model consists of four alternative layers of Simple (S) and Complex (C) units [17]. The S layers employ a Gaussian-shaped function for combining their inputs in order to create the selectivity property in the model and C layers apply a nonlinear operator such as maximum (MAX) to their inputs for building invariance. The basic architecture of the HMAX model has four layers of processing units called *S_1_*, *C_1_*, *S_2_* and *C_2_* which the selectivity and invariance rise as the layers progress along the hierarchical structure of the model. These layers of processing units imitate the behavior of cells from V1 to IT cortex. In the following paragraphs, the functional details of each layer are described.

The lowest layer of the HMAX model, named *S_1_*, receives a gray-value image as its input. Afterward, this input image is applied to a set of edge detector filters. These filters are built based on the Gabor function [37]. Gabor filter fit very well the receptive field weight functions found in simple cells in primary visual cortex [38]. The following equations describe two dimensional Gabor filter.
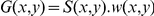



Where s(x, y) is a sinusoidal function called the carrier, and w(x, y) is a two dimensional Gaussian-shaped function, called the envelope.
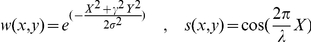


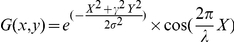


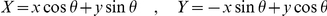



The parameters of Gabor function are defined as follow; 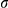 width of Gaussian function, 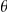 Gaussian orientation, 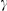 aspect ratio and 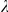 wavelength. In the HMAX model input image is analyzed by a pyramid of filters in different sizes and orientations. The filter sizes start from 7*7 to 37*37 by the steps of 2, therefore, they come in 16 different sizes. The orientations take four angles 0°, 45°, 90° and 135°. Thus, the complete pyramid consists of 4*16 = 64 filters (4 orientations and 16 sizes). These set of filters are divided into 8 bands and each band has a specific size and parameters for the filter window. The parameters of all bands are represented in Table 1 in [17]. Applying this pack of filters to an input image in *S_1_* layer, yield 64 filtered images as *S_1_* layer outputs.

**Table 1:** Image data sets [1], [3], [4].

Data sets	Number of images	sizes (pixel)
Face	435	Different sizes
Face-easy	435	Different sizes
Car-side	123	300*190
Airplane	800	Different sizes
Motorcycle	800	Different sizes
Car (rear)	526	360*240
Leaf	186	896*592
Leopard	200	Different sizes

**Figure 1:**
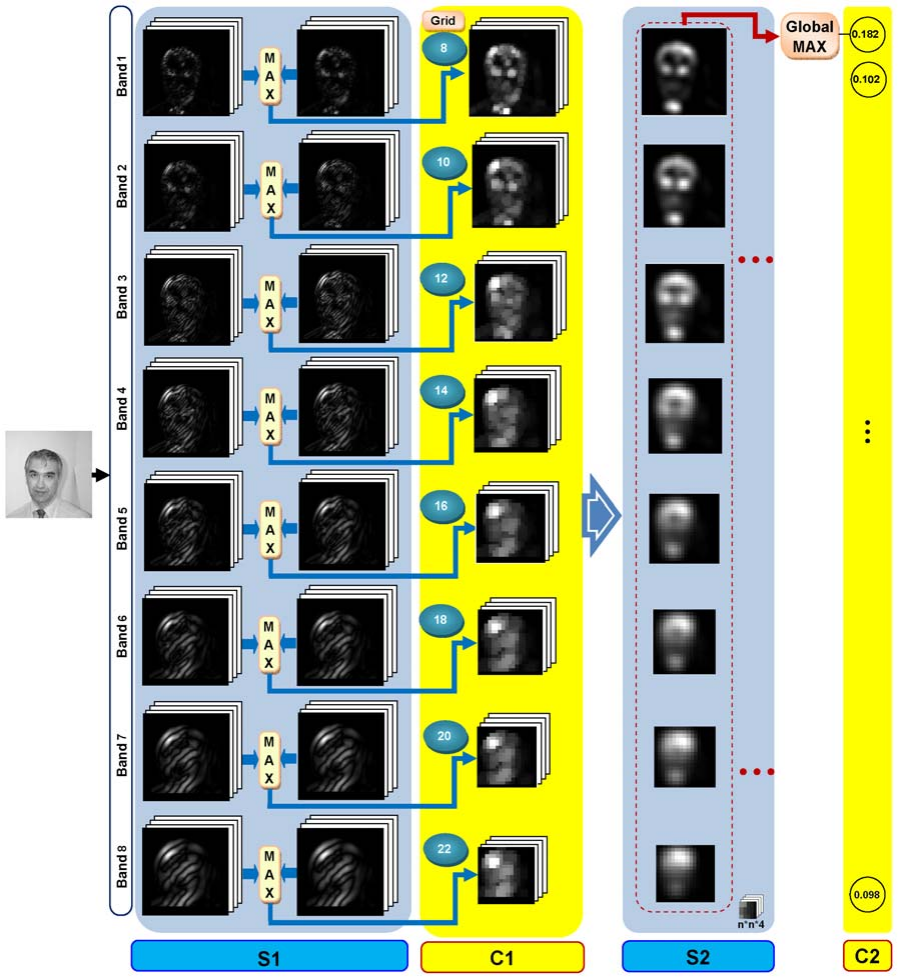
The architecture of the HMAX model. (*S_1_*), In this layer an input image is analyzed with a pyramid of filters (16 filter sizes×4 orientations = 64) and finally 64 filtered images are produced as *S_1_* outputs for an input image. (All filtered images shown in *S_1_* column are only for the orientation 45° for all filter sizes). (*C_1_*), In this layer, the local maximum between 2 adjacent scales with the same orientation is taken.(*S_2_*), The Euclidean distances between stored prototypes, which are obtained in the learning stage, and new input is calculated. This process occurs for all bands in *C_1_* and as a result, *S_2_* maps are obtained. (*C_2_*), The global maximum is computed over all *S_2_* responses in all positions and scales in this layer.

The outputs of *S_1_* are sent to the *C_1_* layer. The behavior of *C_1_* layer is analogous to the complex cells in the primary visual cortex. These types of cells are shown to be position and size invariance within their receptive filed. This property was simply provided by using a maximum operation in *C_1_* layer [10]. The outputs of *C_1_* layer is attained by applying the maximum operator to outputs of *S_1_* layer. Conceptually, the maximum of two adjacent filter sizes in *S_1_* (e.g. 7*7 and 9*9 with the same orientations) is calculated with the maximum operator in order to create some position and size invariance features from *S_1_* to *C_1_*. Therefore, by taking maximum over outputs of *S_1_* layer, we have 32 maps in *C_1_* layer (4 orientations and 8 bands). The next stage in *C_1_* is computing the maximum value in a grid with the cells of size *N* ^Σ^ * *N* ^Σ^ over each map in *C_1_*. The size of *N* ^Σ^ starts from 8 in band1 to 22 in band8 with the overlap of Δ*_s_*. Figure 1 demonstrates the results of this process. In the runtime the Euclidean distance between prototypes which are obtained in the learning stage and new input is calculated. This process occurs for all bands in *C_1_* and as a result, *S_2_* maps are obtained. The *C_2_* layer is the final processing stage in the HMAX model. In this layer the global maximum is taken over all *S_2_* responses in all position and scales. The output of *C_2_* layer is a vector with the length of the number of patches (features) for an input image. The *C_2_* responses then are applied to a classifier such as a Supported Vector Machine (SVM). In the learning stage a large number of patches are extracted in random positions from *C_1_* activations of training images. The range of patch sizes is 4*4, 8*8, 12*12, and 16*16 in all four orientations.

### Genetic Algorithms Introduction

Genetic algorithms (GA) are a family of computational models which are inspired by evolutionary procedure in biological systems. These algorithms are a general adaptive optimization search technique firstly proposed by [39]. GA is an iterative procedure that works with a constant-sized of individuals called population. Each individual of this population present a solution in the search space (the search space contains all possible solutions). Theoretically, it is proved that the GA obtains the best individual as the optimal solution after infinite iterative computations [40]. The GA can deal with those problems that have large search spaces, and have more chance to find the optimal solution than other conventional algorithms.

**Figure 2:**
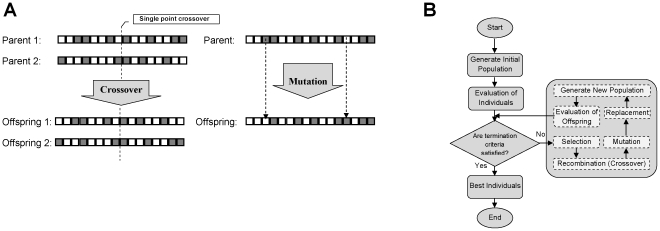
Genetic operations and evolutionary cycle. (A), The crossover and mutation operation. (B),The evolutionary of genetic algorithm.

The GA initially generates a population of individuals. Each individual consists a series of genes. The quality of each individual is assessed based on some criterion and its fitness value is computed. This process is done by a fitness function. Individuals are selected based on their fitness values to produce a new population. There are various methods for selecting individuals. The simplest one was proposed by [39] and is proportional with the probability of individuals' fitness. Several other selecting methods are Roulette wheel, rank selection (which was used in this study), and Boltzmann selection, etc. This procedure eventually leads to the selection of high performing individuals for producing the new population. The fitter individuals have a higher chance to be selected for recombination. Selecting process cannot solely add any new individual to the population; this is done by using two main genetic operators, crossover and mutation. Crossover is a randomly selecting mechanism for exchanging genes between two selected parents in order to create new offspring. Some well-known crossover methods are single point crossover, two point crossover, and uniform crossover –the latter is employed in this work. One-point crossover is not invariant under changes in the order of patches. When using one-point crossover the assumption is that the order matters, whereas for uniform crossover this is not the case [41]. Generally, it is in not desired. Therefore, uniform crossover was used. One of the benefits of using more crossover points is that the problem space may be searched more thoroughly [42]. Therefore, crossover combines the features of two individuals to create two offspring. The mutation is an operator which allows diversity. During the mutation stage the genes of a selected individual may randomly be changed. For instance, in a binary string, one or more bits (gene) convert their value from 0 to 1 or vice versa in a random position in the string. This operator inserts new information into the population. Offspring replaces the old population using the elitism or diversity replacement strategy and creates a new population in the next generation. This iterative process progresses until the termination criteria are satisfied. Figure 2 is a general illustration of the crossover, mutation, and the GA evolutionary process.

### The proposed model

In this paper, we use a genetic algorithm approach to select more effective features in diverse object recognition tasks. Genetic algorithms generate a powerful method to find nearly the best and most optimized solutions for a complex problem. Sometimes, however, they fail to reach this goal because of falling in a local optimum. Nevertheless, they have been widely used in various applications such as feature selection [23]–[26] and parameter optimization in recent decades [27]–[31].

From the pattern recognition point of view, feature selection is an important issue that can impact on the classification accuracy. The proposed model employs an evolutionary approach for selecting proper features in several object recognition tasks. In our study, extracted patches from *C_1_* activation in the HMAX model are considered as features. In order to select effective features by GA, we should design the chromosome structure, fitness function, and system architecture.

#### Chromosome Structure

**Figure 3:**
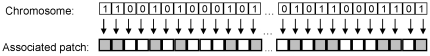
Chromosome structure. A chromosome is a binary string with a length of N to represent the presence or absence of each of the N possible features. Each bit of this binary vector is related to a feature. If the value of a bit is *1*, then it represents the presence of a particular patch in the learning procedure, and if the bit is *0*, it shows this patch does not have any effect on learning process.

The first phase of the proposed model is to design an appropriate structure for chromosomes. We used the binary coding approach to represent a chromosome. Therefore, a chromosome is a binary string with a length of *N* to represent the presence or absence of each of the *N* possible features (patches) by *1* or *0*, respectively. Each bit of this binary vector is related to a feature. If the value of a bit is *1*, then it represents the presence of a particular patch in the learning procedure of the HMAX model; on the other hand, if the bit is *0*, it indicates this patch does not have any effect on the learning process. Figure 3 illustrate the chromosome structure.

In Figure 3, the first row demonstrates the binary structure of a chromosome and the second row shows the patches that participate in the learning phase of the HMAX model (gray squares are patches which participate in learning). The initial population of GA algorithm consists of a pool of these chromosomes.

#### Fitness Function

The chromosomes should be evaluated with an appropriate evaluation function. This is an important phase for making a successful application of GA for a feature selection problem. The chromosome with high fitness value has more chance to be preserved in the next generation. So, choosing a suitable fitness function can have a considerable impact on selecting the appropriate patches. In our research, all data were randomly divided into three subsets including train, evaluation, and test. The fitness function, *ρ,* was the classification performance of the model on the evaluation subset. The GA selects a set of patches that maximize the *ρ* or minimize the *F = 1-ρ*.

#### System Architecture

In order to construct the proposed model, several main steps should be considered.

1. *Extracting a Pool of Patches from C_1_ Activations:* Firstly, the training images are analyzed by a set of Gabor filters and produce *S_1_* outputs. The *S_1_* responses applied to *C_1_* layer and then *C_1_* activations are created. The next stage is patch extraction, in this phase a large number of patches in random positions are extracted from *C_1_* responses in all four orientations (0, 45°, 90°, 135°) and sizes (4, 8, 12, 16). It is important to point out that we only use *Band 2* parameters in Table 1 of [17] to extract patches from *C_1_* responses.
2. *GA Initial Population*: After extracting patches, an initial population of chromosomes is generated by GA. The length of each chromosome is equal to the length of extracted patches in the first step (*N*). Based on the position of *1 s* and *0 s* in the binary string of chromosome structure, some patches are selected from the pool of patches. Figure 3.
3. *Obtaining C_2_ Features:* selected patches are used to create *S_2_* responses and then *C_2_* features for all images (the details of the HMAX model were previously described). let *P* be the number of selected patches, then *C_2_* features for an arbitrary input image will be a vector of length P*1. If we have *K* images, the dimension of *C_2_* matrix will become P*K. Each row of this matrix is associated with a particular patch response.
4. *Evaluation of Chromosomes:* for each chromosome representing selected patches, the classification performance on evaluation subset is calculated using a linear SVM classifier. Each chromosome is evaluated by fitness function *F = 1-ρ*.
5. *Termination criteria:* in this step the termination condition is checked and if the GA meets the criterion, the process ends; otherwise, it moves on to the next generation.
6. *GA operations:* classical GA operators such as selection, crossover, mutation, and replacement are applied to the chromosomes for finding a better solution (best patches).

**Figure 4:**
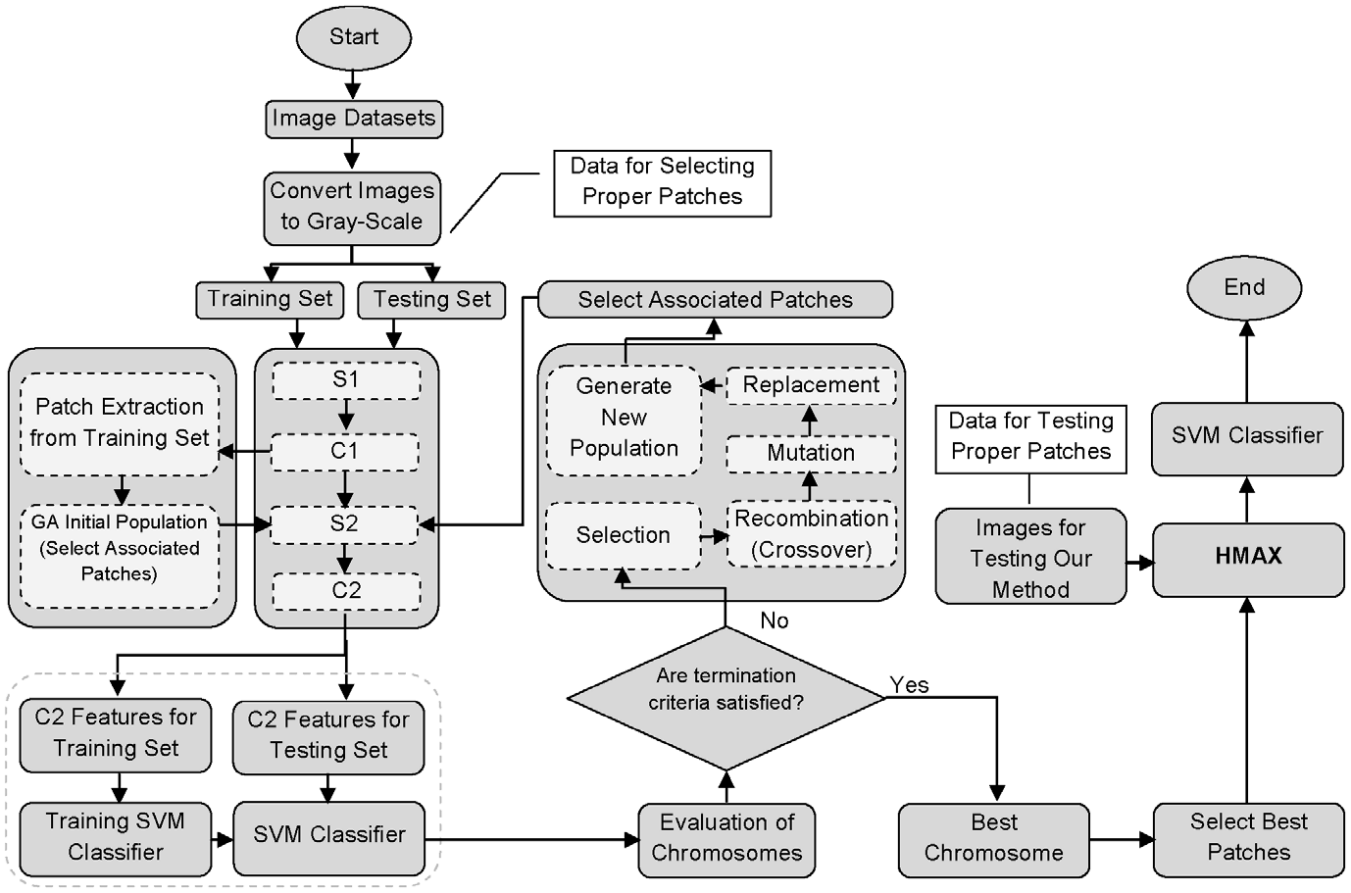
The architecture of the proposed model.

Figure 4 illustrates the whole model structure. After selecting the best group of patches we test them on testing data. It is important to point out that all GA parameters were tuned one time on the evaluation set and no other changes occurred along test experiments.

### Image date sets

In order to evaluate the proposed model, we used a variety of object image data sets.

#### CalTech

We tested the model on five of the databases, i.e., the face, motorcycle, rear-car, and airplane data sets from [1] as well as the leaf data set from [3] and leopard, car-side and face-easy from [4]. On these data sets, we followed the same way as in the corresponding studies. Object classes were used as target images and a background folder as negative examples. Each of these data sets contains different number of images in various sizes; Table 1 shows the sizes and the number of images for each data set. In spite of many serious concerns raised about the Caltech-101 data set [16], [43], that test is still widely used in the object recognition community and thus most state-of-the-art algorithms have reported accuracy on Caltech-101 in the literature [44]–[49]. Nonetheless, in order to show the effectiveness of the proposed model, it was also tested on some more challenging and updated image date sets.

#### GRAZ

**Figure 5:**
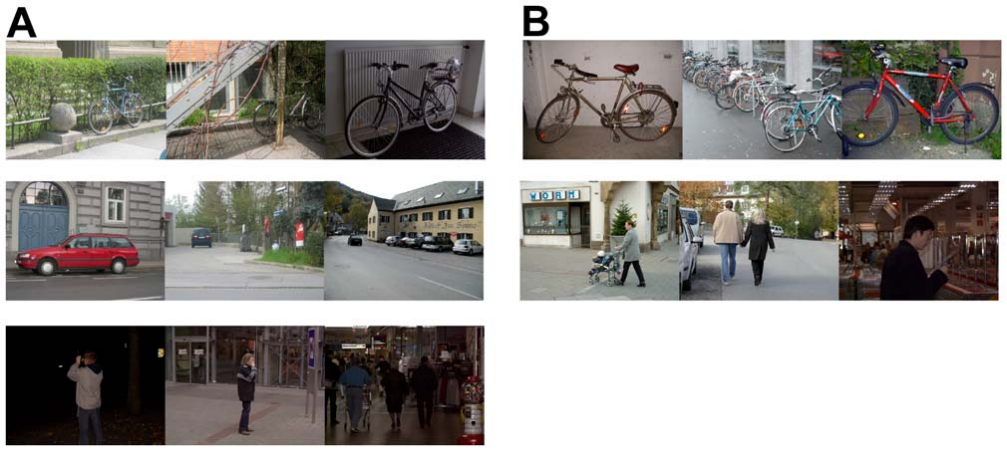
Some image samples of GRAZ data set. (A), Sample images of GRAZ-02, bike, car, and person images. (B), Sample images of GRAZ-01, bike and person images.

The GRAZ database which is part of the PASCAL Object Recognition Database Collection and was built by Opelt et al [35], consists of two challenging data sets. First, The GRAZ-01 data set contains three classes which are varied in locations, scales and viewpoints. Next, the GRAZ-02 data set was built with the purpose of increasing the independence of background context for categorization. In addition, they increased the complexity of object appearances and car images also were added as a new category. Figure 5 shows some samples of each object category for both data sets. For the GRAZ data set, we follow the way in [35].

### Experiential design

We firstly evaluated the performance of the proposed model in the object present/absent experiment using several object classes from CalTech data set. In this experiment, each data set was randomly divided into three subsets with equal number of images, the first subset, called training examples, was used for extracting a pool of patches, the second subset was the evaluation data, this set of images was used for GA patch selection procedure, and the last subset was used for testing the proposed model. We also investigated the effect of the number of training examples on the recognition performance. In this experiment, the model was trained on various numbers of positive training images (1, 3, 6, 15, 30, and 40) and 50 negative training images to compare with [17]. The number of testing images was 50 positive images and 50 negative images. All images were converted to gray-scale and resized to 140 pixels in height and the width resized proportionally to height variation. All experiments were tested on 10 random runs and the average and standard deviation were reported. In order to compare the influence of our selected patches and randomly extracted patches on the recognition performance, the proposed model was run on different data sets with various numbers of patches. The model selected *P* effective patches from *N* randomly extracted patches by the HMAX model (the number of *P* is variable and depends on the *N* and task). The following section discusses the experimental results. We report the error rate at equilibrium point as the accuracy measure for the performances in all experiments. For the sake of proving that the difference between the performances of the HMAX model and the proposed model are statistically significant, we used two non-parametric statistical tests, i.e. Wilcoxon rank sum [50] and two-Sample Kolmogorov-Smirnov test [51].

For the GRAZ-01 data set, 100 positive and 100 negative images were randomly selected as training samples and 50 other positive and 50 negative images were selected as testing samples, as used in [35]. We also followed the same way in [35] for GRAZ-02, 150 positive and 150 negative images were selected at random as training samples and 75 other positive and 75 negative images were randomly selected as testing samples. All the experiments were run 20 times and the average ROC area under curve (AUC) and Equal-Error rate (EER, which means the detection rate at equal-error-rate of the ROC curve.) were reported as performance measurement. A comparison was drawn on these data sets between the proposed model and some other feature extraction methods: Moment Invariants, SIFT, SM, Basic moment in [35] and EBIM in [36].

## Results

In this section, we report the results of several classification experiments performed on different object classes. Table 2 compares the results of the proposed model with the HMAX model for CalTech image data sets. This experiment is a simple object absent/present one. We divided each object category to three equal-sized parts, one for training, another for evaluation and the third one for testing the HMAX model and the proposed model. We used linear SVM classifier in all experiments. The results in Table 2 indicate that the proposed model outperforms the HMAX model in different object recognition tasks.

**Table 2:** Comparison between the HMAX and the proposed model for different image data sets.

	Performance of HMAX [17]	Performance of Proposed Model	Statistical Significance	
Data sets	Eq pt	Eq pt	Two-Sample Kolmogorov-Smirnov Test [51]	Wilcoxon rank sum test [50]
			p-value	p-value
Airplane	94.9	97.2	0.3129	0.2853
Face	98.1	98.7	0.3127	0.0949
Leaf	95.9	98.8	0.3129	0.1175
Motor	97.4	99.3	0.0310	0.0134
Car (rear)	99.8	99.8	1.0	0.9095

**Figure 6:**
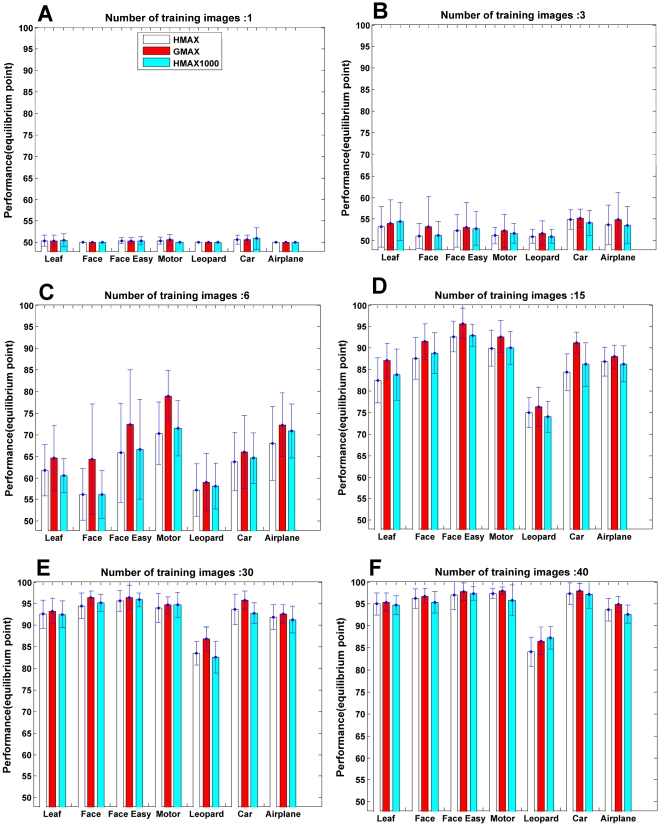
The performance which was obtained with different number of training examples for our model and the HMAX model. (A), The performances for one positive training image (all other image sets, negative training, positive test and negative test consist of 50 images), in most data sets the performance for all three cases are almost equal. (B, C), The performance for 3 and 6 training examples respectively. (D, E, F), Performance of the models for 15, 30, and 40 training images respectively. White bars show the performance of the HMAX model with *P* randomly extracted patches and the red bars illustrate the proposed model performance, cyan bars show the performance of the HMAX model for 1000 patches.

We also studied the influence of different number training examples on the resulting classification performance. Figure 6 makes a comparison between the performance of the proposed model and the HMAX model for different numbers of training images (1, 3, 6, 15, 30, 40) using the SVM classifier. In this experiment, several object classes such as Face, Leopard, Airplane, Leaf, Car-Rear, Motorcycle and Face-Easy from Caltech data set were used as target (positive) images. Each object category was randomly divided into three subsets as was described in the experimental design. The background folder of this data set also was used as the negative images.

The experiment was carried out as follows: Firstly, the HMAX model was run with 1000 randomly extracted patches. Then, the proper patches (*P*) were selected and the performance of them was calculated on testing images. Finally, for fair comparison, the same number of patches (*P*<1000) was randomly extracted by the HMAX model and the recognition performance was computed on the same testing images that used for the proposed model. Figure 6 compares the results of the HMAX model with the proposed model. The white bars show the performance of the HMAX model with *P* randomly extracted patches and the red bars point out the proposed model performance. These two bars (white and red) represent a fair comparison between the HMAX model and ours. In addition to this, we ran the HMAX model with 1000 random patches to compare the HMAX model with the proposed model for another level of comparison; the results of this experiment are represented with cyan bars. In both cases (the HMAX model with 1000 patches and the HMAX model with *P* patches), the proposed model shows significant improvement in recognition performance. Figure 6A demonstrates the performances for one positive training image (all other image sets, i.e. negative training, positive test and negative test consist of 50 images). In most data sets the performance for all three cases are almost equal because we have only one training. As the number of training images increase (i.e. 6 and 15), the difference between the performance of the proposed model and two other cases becomes more considerable, Figure 6C, 6D.

It illustrates that the proposed model achieves a recognition performance comparable to [17] on a few training examples (less than 30). While the number of training images reaches 30 and 40, as it is predictable, this difference goes down, since the more training images there are, a better performance is achievable.

**Figure 7:**
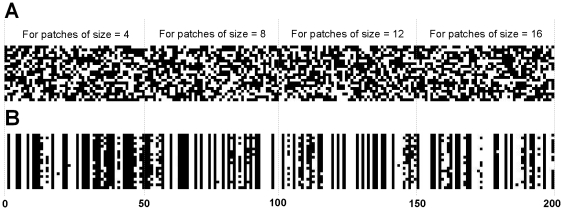
Final and initial population of GA. (A), The initial population of GA. (B), The final population of GA (each white pixel shows the presence of a patch in learning procedure).

Our goal was to select proper patches in various object recognition tasks. For this, we extracted a large number of patches in different sizes (4, 8, 12, and 16) from training images. Then, the most effective patches were selected. An important question is: which patches of what sizes have more influence on the recognition performance? We explored the final population of selected patches and best individuals of GA to answer this question. Briefly, The GA starts with an initial population and terminates by generating the fittest population and individuals. The length of each chromosome in the final population in our research is equal to the number of randomly extracted patches and the size of population is 20. For instance, if the number of extracted patches for each size is 50, then the chromosome is a binary vector of size 1*200 and each bit is associated with the presence or absence of a particular patch in the learning phase (see the proposed model). Therefore, the dimension of population matrix will be 20*200. The position of *1 s* and *0 s* in the final population along with the fittest chromosome can represent which patches have been selected more than others. In Figure 7 the population matrix is shown as an image for the sake of visualization, Figure 7A represents initial population and Figure 7B shows the final population (this is the final population for face images). It depicts the diversity of final population. Each white pixel indicates the presence of a patch and each black pixel the absence of a patch. Each row of this matrix is a chromosome that represents which patches are selected. It is clear from Figure 7 that some patches (white columns) are more informative than others and have the same position in most chromosomes, so, they are selected more than other patches. It can also be seen that some patches from particular sizes have been selected more than other patch sizes (i.e. in Figure 7 the number of patches of size 8 and 12 in the final population for face images are more than other sizes).

**Figure 8:**
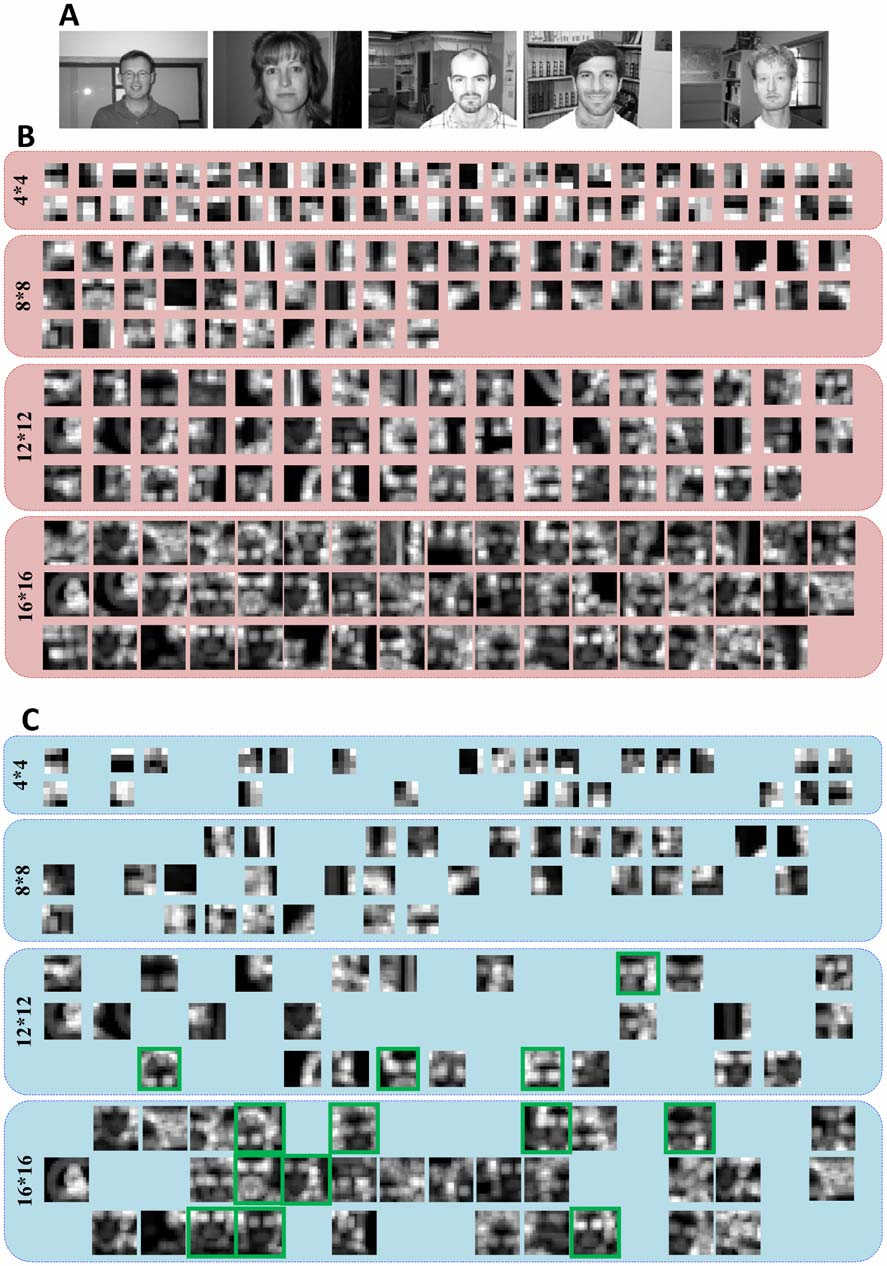
Selected patches. (A), Examples of face images from Caltech image data sets. (B), Randomly extracted patches by the HMAX model. (C), Informative patches which are selected by our model.

The extracted patches by the HMAX model are from random position in an image. So, these patches may come from background or other irrelevant objects rather than the target object. The *C_2_* features that are obtained from these patches are not very useful for recognizing a target object. They may also make the feature space more complex for classification. The proposed model selects those patches which are more informative. Figure 8 shows random patches extracted by the HMAX model and selected patches by the proposed model for face images. Figure 8A shows samples of input images. We extracted 50 patches for each size and then selected the most informative ones. Each row from the top to the bottom in Figure 8B represents patches from a particular size (4, 8, 12, and 16). It can be seen from Figure 8C that some selected patches are a part of a target image such as eye, a part of a face and in some cases a complete face (they are depicted by green frames). Since, the HMAX model uses a template matching approach and computes the distance between sorted prototypes and input image, therefore, patches that are parts of target object are more informative than other patches in recognizing an object, because the matching degree between these patches and target image is much more than non-discriminating patches.

**Figure 9:**
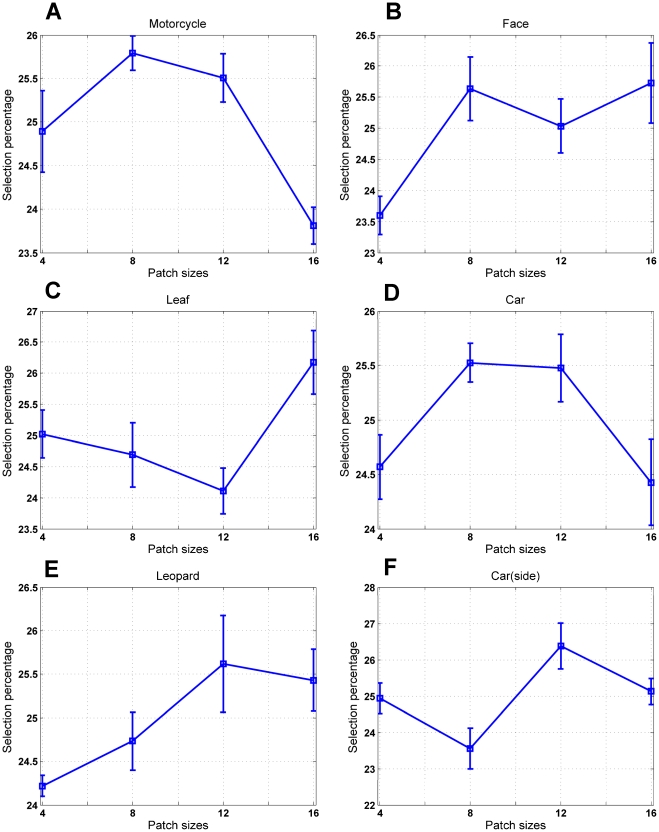
Selection percentage for different patch sizes. (A), The selection percentage for motorcycle images. (B), The selection percentage for face images. (C, D, E, F), The selection percentage for leaf, car, leopard and car(side) data sets respectively.

We can see that some selected patches (i.e. from lager sizes particularly in face images) are the most important parts of the target objects; thus, the *C_2_* features which are formed from these patches are very discriminative and can differentiate between the target objects and other distractors. For instance, in face images, patches of size 8 and 16 are selected more than other patch sizes. But, are the same patch sizes selected more than others for other object categories? For further study, we explored the number of selected patches of each size for different object images. In this experiment, we extracted *N* random patches from each training set of different object classes (*N* = 1000) and then selected *P* patches. We then calculated the selection percentage of each patch size 4, 8, 12, 16. Figure 9 illustrates the selection percentage of each patch size for different object images. The results are the average of 10 random runs.

It can be seen that some particular patch sizes in different object images have been picked up more frequently. For example, in face images patches of size 8 and 16 (and 12 with a small difference) are more effective than other sizes. These sizes can usually cover some important parts of a face, so they are useful in recognizing the target images. In motorcycle images, patches with the size of 8 and 12 have been selected more than others or in leopard class selected patches are mostly of size 12 and 16. Therefore, it shows that some particular patch sizes in various object recognition tasks have more influence than others.

**Figure 10:**
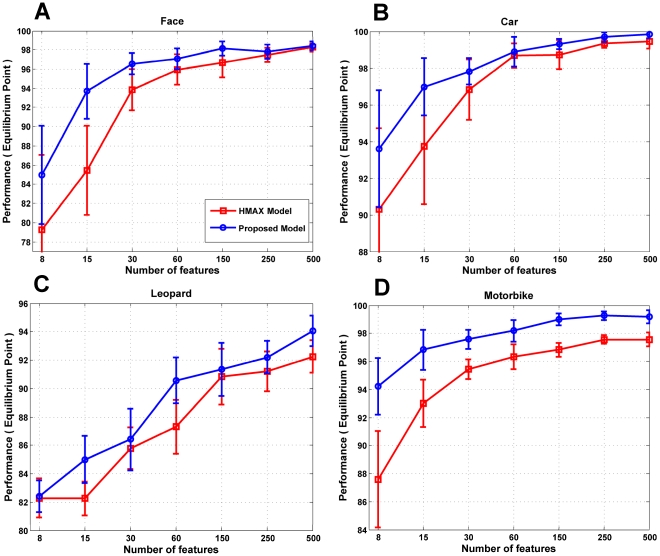
Recognition performance for different number of features. (A), Comparison between the HMAX model and the proposed model for different numbers of patches for face images.(B, C, D), The performance for Car, Leopard and Motorbike images respectively for the proposed model and HMAX.

The number of *C_2_* features directly depends on the number of patches which are extracted during the training phase in the HMAX model or selected in the proposed model. Here, we compared the performance of the proposed model when different number of patches was used with the HMAX model in several categorization tasks. We considered four object images from Caltech data sets, Face, Leopard, Motorcycle and Car-rear. Firstly, we extracted a set of random patches from training images and then selected a subset of them. Then, the performance of selected patches and random patches were computed on the same testing images. Figure 10 makes a comparison on several object classes for different number of features between the HMAX model and the proposed model. In all cases, the proposed model outperforms the HMAX model. As it is mentioned above, some irrelevant patches that come from other objects can decrease the recognition performance and may cause complexity in features space. Eliminating these patches can improve the quality of *C_2_* features and consequently the performance of the model will be increased, even by making use of a very small number of patches.

As Figure 10 shows, the performance of the proposed model for face images is approximately 94% for 15 patches, whereas it is about 86% for the HMAX model. The proposed model has reached 97% in performance with 60 patches while the HMAX model achieved this amount with almost 250 patches. An important result is that despite the fewer number of patches in the proposed model the evolutionary feature selection mechanism is able to find good results in different object recognition tasks.

The proposed model was also run on GRAZ data set for making a comparison with other feature extraction methods. Table 3 shows the results of this comparison on GARZ-01 data set.

**Table 3:** Comparison between several feature extraction methods on Graz-01.

	Data sets			
Methods	Bike		Person	
	EER	AUC	EER	AUC
Moment Invariants [35]	73.5	76.5	63.0	68.7
SIFT [35]	78.0	86.5	76.5	80.8
SM [35]	83.5	89.6	56.5	59.1
EBIM [36]	84.1	90.5	86.0	91.8
HMAX	75.5	85.2	74.8	81.5
**The Proposed Model**	**80.2**	**88.5**	**84**	**90.8**

The measures are EER (Equal-Error Rate) and AUC (ROC-Area Under Curve). (Our reported results are the average of 20 independent runs).

**Figure 11:**
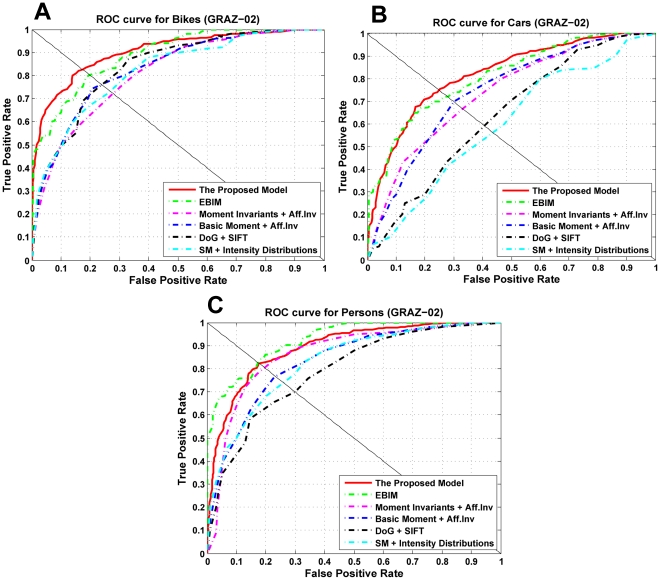
Comparison of several approaches on GRAZ-02 database. (A), ROC curves for Bike images. (B,C), ROC curves for Car and Person images respectively.

Table 4 and Figure 11 depict the obtained results for the same comparison on GRAZ-02 data set.

**Table 4:** Comparison between several feature extraction methods on Graz-02.

	Data sets		
Methods	Bike	Person	Car
Moment Invariants [35]	72.5	81.1	67.0
Basic moment [35]	76.5	77.2	70.2
SIFT [35]	76.4	10.0	68.9
SM [35]	74.5	74.1	56.5
EBIM [36]	80.8	83.2	72.2
HMAX	81	80	72
J. Mutch et. al. [46]	80.5	81.7	70.1
**The Proposed Model**	**82.6**	**82.3**	**75.6**

The measures are EER (Equal-Error Rate). (Our reported results are the average of 20 independent runs).

## Discussion

Several brain areas in a primate visual cortex are involved in object recognition. Many years of research in neurophysiology and neuroscience have disclosed substantial amount of information about visual object processing [18], [19]. One commonly accepted property is the hierarchical processing of objects in the visual cortex. According to this, different visual features are selected and analyzed in different stages along the hierarchy. In the earliest processing stages, from retina to LGN then to primary visual cortex V1, the object image is represented by a set of simple features such as oriented bars and edges. After these primary stages, some more complex features are represented in intermediate stages by combining simple ones in V2 and V4 and finally in IT cortex. Based on these data, a computational object recognition model was proposed by [10], [17]. In this model a set of hierarchical units are used to create a large set of features from images in order to categorize target objects in a scene.

Briefly, after early processing stages in this model (*S_1_, C_1_*), a set of patches are randomly extracted from an input image. Since, the target object (i.e. face) does not occupy a large area in an image, therefore, this random mechanism may extract patches from irrelevant parts of an image rather than target object. As it is demonstrated in Figure 8, we found that some *C_2_* features which are created based on these random patches could be due to those image parts that belong to the target object (i.e. eyes, as a part of a face or in some cases a complete face in face images) and some other *C_2_* features are related to nondiscriminating parts such as background and other objects. These nondiscriminating patches can decrease the recognition performance.

In this paper, a biologically motivated object recognition model is proposed based on the HMAX model. The random mechanism of patch extraction in the HMAX model is considered as a limitation. To overcome this problem, we extended the HMAX model by modifying the patch extraction mechanism using a genetic algorithm approach. The main idea behind the proposed model is that an efficient and biologically inspired feature selection mechanism can substantially reduce the dimension of feature space in the higher levels of processing for the sake of better classification rate. An important result is the fact that despite the fewer number of patches in the proposed model are used it is still able to find better results in different object recognition tasks which reveals the ability of our proposed mechanism in extracting informative patches. After selecting a set of patches in the proposed model, we have studied the final population of patches and found that some particular patches are selected more than others along several generations. It was found from the experimental results that most of these selected patches are parts of target objects.. We compared the recognition results obtained using optimal features with those obtained using the HMAX random mechanism. Our results showed that the proposed model outperforms the HMAX model in different object recognition tasks with fewer numbers of patches. From the feature selection point of view, in visual processing, our results have two significant implications: First, they show that visual features, which are more informative, are prevalently come from target objects. Second, they show that the model can achieve a higher recognition performance (or the same recognition performance in a few cases) with fewer numbers of patches.

It is probable that the visual system uses a similar mechanism to recognize objects. Biologically evidence suggests that both genetic factors and visual experience can determine the functional properties of units in the visual cortex [32], [33].The development of human visual system is mainly influenced by genetic factors and visual experiences. However, how genetic factors can affect neural activations in the visual cortex is not completely clear. Recently, an interesting study has been done on twins' visual cortex using functional magnetic resonance imaging (fMRI) [33]. The focus of this study was on the neural activity patterns in twins' visual cortex which were related with some objects categories (such as faces and places) to appraise the role of genetics in determining the neural activity patterns associated with these visual categories. Their results demonstrated that these patterns are significantly more similar in monozygotic twins than dizygotic twins. These results simply suggest that genetics may play a significant role in the plasticity of visual cortex and cortical responses to some object categories.

Since we use GA in the training procedure to select informative patches, one may think that the proposed model is time-consuming. Although the training phase may take pretty long computational time, the testing phase is much faster, because we have only a few numbers of patches though differentiating ones. Furthermore, an analogy can be drawn between this time-consuming process of GA and the development of neural circuits involved in a visual recognition task throughout years which is also a time-consuming process. Finally, it worth saying that compared to the complexity of the human visual system, our model ,like all of the other biologically inspired object recognition models, may describe only a little of capabilities and flexibility of this admirable system.
